# Chemotherapy‐Induced Peripheral Polyneuropathy in Pediatric Acute Lymphoblastic Leukemia: A Case Report on Manifestation, Management, and Outcome

**DOI:** 10.1002/cnr2.70293

**Published:** 2025-08-20

**Authors:** Eman Al Mattar, Sondus Al Sharidah, Omnia A. Hashem

**Affiliations:** ^1^ Pediatric Hematology/Oncology and Stem Cell Transplant Unit NBK Specialized Children's Hospital Kuwait City Kuwait

**Keywords:** chemotherapy‐induced peripheral polyneuropathy (CIPN), pediatric acute lymphoblastic leukemia (ALL), quality of life

## Abstract

**Background:**

Chemotherapy‐induced peripheral polyneuropathy (CIPN) is a debilitating side effect of cancer treatment such as in acute lymphoblastic leukemia (ALL). Characterized by the dysfunction of the peripheral nervous system, CIPN can occur in 90% of pediatric patients, significantly impairing their quality of life. In most cases, it necessitates the modification of standard chemotherapy regimens, which can compromise the treatment efficacy and increase the risk of relapse.

**Case:**

We report a 16‐year‐old boy with ALL who developed CIPN during induction therapy. A multidisciplinary, individualized treatment approach combined with regular follow‐ups ensured optimal management. The patient achieved complete remission with significant improvement in mobility and neurological functions.

**Conclusions:**

Early recognition and comprehensive management of CIPN in pediatric ALL patients are essential to optimizing outcomes while maintaining the overall efficacy of chemotherapy.

## 1 Introduction

Acute lymphoblastic leukemia (ALL) is a hematologic malignancy characterized by uncontrolled proliferation of B‐ or T‐lymphocyte progenitor cells, leading to bone marrow infiltration and suppression of normal hematopoiesis. In Kuwait, 50–70 new cases of leukemia are diagnosed annually. Advances with intensive chemotherapy regimens have resulted in complete remission in 85%–90% of ALL patients, with long‐term survival rates averaging 80% [1]. However, these treatments have also increased the risk of complications, such as chemotherapy‐induced peripheral polyneuropathy (CIPN), which affects up to 90% of pediatric patients and survivors [2, 3], posing a significant challenge in cancer management. CIPN can manifest as either pure sensory and painful neuropathy or sensorimotor neuropathy with or without involvement of the autonomous nervous system and can even develop after a single drug application [4]. Severe CIPN can lead to long‐term dysfunction and a significant decline in the quality of life, often necessitating dose reductions or discontinuation, potentially impacting patient survival [5]. Hence, its early recognition and management are essential to mitigate such severe outcomes.

Among various drugs used in the treatment of ALL, vincristine is well known for its neurotoxicity, often leading to vincristine‐induced peripheral polyneuropathy (VIPN) with consequent reductions, delays, or even discontinuation of vincristine, along with significant pain, disability, and a reduced quality of life for patients [6]. Despite dose adjustments, VIPN can persist for 8–12 months, especially in older children with severe manifestations [7, 8]. Moreover, since vincristine is a cornerstone agent of pediatric cancer treatment, its discontinuation raises concerns regarding overall treatment success.

We present a case of a 16‐year‐old boy with pediatric ALL complicated by CIPN, which required modification of vincristine chemotherapy from the standard regimen. This case highlights the challenges of CIPN management and strategies to optimize favorable patient outcomes.

## 2 Case

A 16‐year‐old boy was referred to our clinic, Pediatric Hematology/Oncology and Stem Cell Transplant Unit at NBK Specialized Children's Hospital, Kuwait, in November 2023. He presented with a two‐week history of intermittent headaches and asthenia, followed by 4 days of epistaxis. On examination, he appeared pale but alert and oriented, with no history of fever, weight loss, or altered bowel habits. Initial investigations with complete blood count (CBC) and blood film analysis revealed severe anemia, absolute neutropenia, lymphocytosis, and the presence of 35% blast cells (Table 1). His coagulation profile also indicated prolongation. After receiving blood and platelet transfusions, his symptoms were controlled, and further investigations were planned. Family history revealed no significant findings, except for the father's hypertension and use of ongoing oral hypoglycemic medication.

**TABLE 1:** Hematological parameters before chemotherapy.

Test	Value	Reference range
Hemoglobin	2.9 g/L	13–17 g/L
WBC	2.5 × 10^9^/L	4–10 × 10^9^/L
Neutrophils	10%	40%–80%
Lymphocytes	44%	20%–40%
Platelets	14 × 10^9^/L	150–410 × 10^9^/L
Blast cells	~35%	0%

**FIGURE 1:**
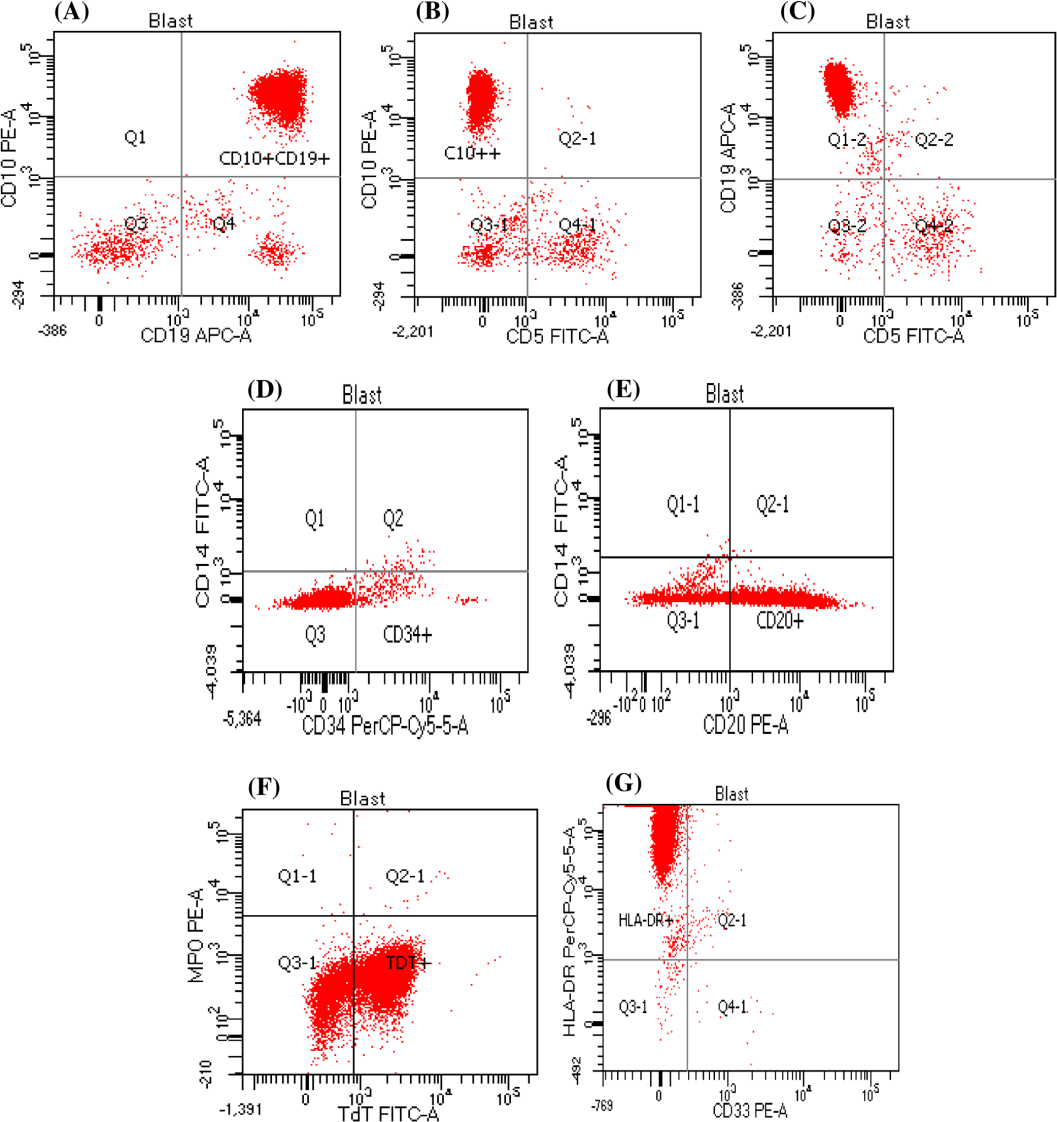
Quantitative flow cytometric analysis of peripheral blood at diagnosis. (A‐C) Dot plots from a 43.2% blast population show co‐expression of CD19 and CD10 with majority of the gated population located in the upper right quadrant (Q2), indicating the presence of CD19 + CD10+ cells (88.9%) (A); co‐expression of CD5 and CD10 with most events in Q1‐1 quadrant indicating, CD5‐CD10+ expression (88.8%) (B); co‐expression of CD5 and CD19 with majority of events in Q1‐2, indicating CD5‐CD19+ expression (92.4%) (C). Scattered events in remaining quadrants likely represent residual normal B‐cells or background. (D‐E) Dot plots from a 40% blast population show negative expressions of CD34 (3.9%) in Q4 and CD14 (0.0%) in Q1 (D) and positive expression of CD20 (75.6%) in Q4‐1 (E). (F) Dot plot from a 54.3% blast population shows positive expression of TdT (73.8%) in Q4‐1 and negative expression of myeloperoxidase (MPO) (0.1%) in Q1‐1. (G) Dot plot from a 42.6% blast population shows positive expression of HLA‐DR (97.9%) in Q1‐1 and negative expression of CD33 (0.2%) in Q4‐1. CD14‐, MPO‐, CD33‐ expressions ruled out myeloid lineage involvement. CD19+, CD10+, CD5‐ expression supported precusor B‐cell acute lymphoblastic leukemia (ALL). HLA‐DR+ expression also supported blast phenotype. CD20+ and CD34‐ suggested a more mature precursor B‐cell phenotype. TdT+ ruled out Burkitt‐type and confirmed lymphoid blast population. This immunophenotypic profile confirmed the diagnosis of B‐cell ALL.

Flow cytometric analysis of the patient's peripheral blood showed that ~50% of cells were gated in the lymphoid/blast region. Further, a high percentage of positive expression for CD10 (88.8%), CD19 (92.4%), CD20 (75.6%), HLA‐DR (97.9%), and Terminal Deoxynucleotidyl Transferase (TdT) (73.8%) confirmed the diagnosis of B‐cell ALL (Figure 1). Supporting data with CD45/SSC‐A dot plots are also shown in Figure S1–S4 (in Supporting Information). The patient was managed with treatment as per the UK (United Kingdom) ALL 2011 Regimen B protocol [9] (Table 2).

**TABLE 2:** Treatment as per UK ALL 2011 regimen B protocol [9].

Drug	Route	Dosage	Frequency	Duration	Total doses
Induction therapy					
Methotrexate	Intrathecal	12 mg/dose	On days 1, 8, and 28	4 weeks	3
Daunorubicin	Intravenous	25 mg/m^2^/dose = 46.5 mg/dose	Weekly	4 weeks	4
Vincristine	Intravenous	1.5 mg/m^2^/dose = 2 mg/dose	Weekly	5 weeks	5
Dexamethasone	Per Os	6 mg/m^2^/day = 11 mg/day	Daily	4 weeks	28
6‐Mercaptopurine	Per Os	75 mg/m^2^/day = 110 mg/day	Daily	1 week	7
Berlin‐Frankfurt‐Munster (BFM) consolidation therapy					
Cyclophoshamide	Intravenous	1000 mg/m^2^/dose = 1780 mg/dose	On days 1 and 15	2 weeks	2
6‐Mercaptopurine	Per Os	75 mg/m^2^/day = 110 mg/day	Daily	4 weeks	28
Cytarabine SC	Intravenous	75 mg/m^2^/day = 133.5 mg/day	4 pulses of 4 days each	4 weeks	16
Interim maintenance					
Methotrexate	Intrathecal	12 mg/dose	On days 1, 8, and 28	4 weeks	3
Methotrexate	Per Os	20 mg/m^2^/dose = 35.6 mg/dose	Weekly	7 weeks	7
6‐Mercaptopurine	Per Os	75 mg/m^2^/day = 110 mg/day	Daily	8 weeks	56

**FIGURE 2:**
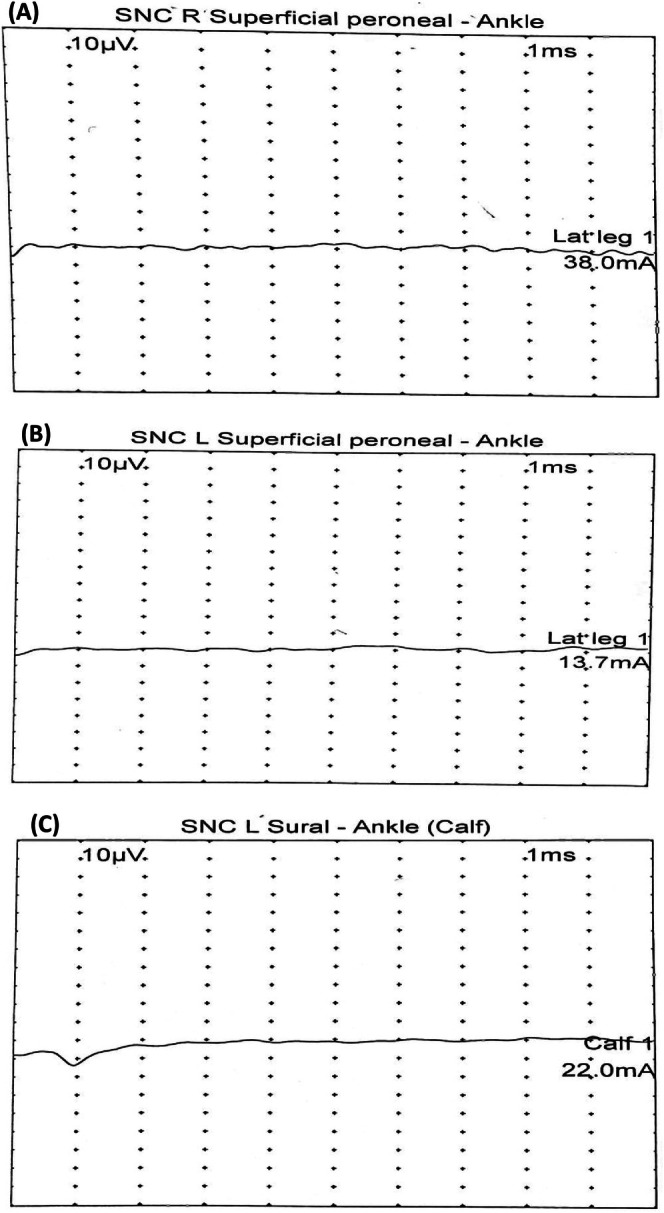
Electromyography/nerve conduction studies. The electromyography/nerve conduction studies of lower limbs demonstrated the absence of response in the right superficial peroneal nerve in ankle (A), absence of response in the left superficial peroneal sensory nerve in ankle (B), and absence of response in the left sural sensory nerve in ankle (C).

**FIGURE 3:**
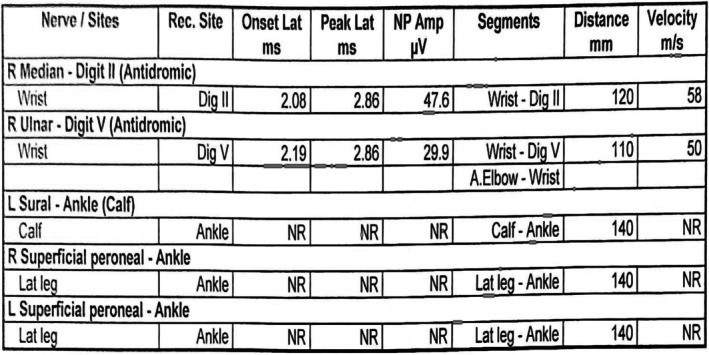
Sensory nerve conduction study. The results showed normal responses with normal onset latency, peak latency, amplitude and conduction velocity in the right median and ulnar nerves. In contrast, no response was recorded in the left sural and bilateral superficial peroneal nerves, suggesting possible sensory neuropathy in the lower limbs.

**FIGURE 4:**
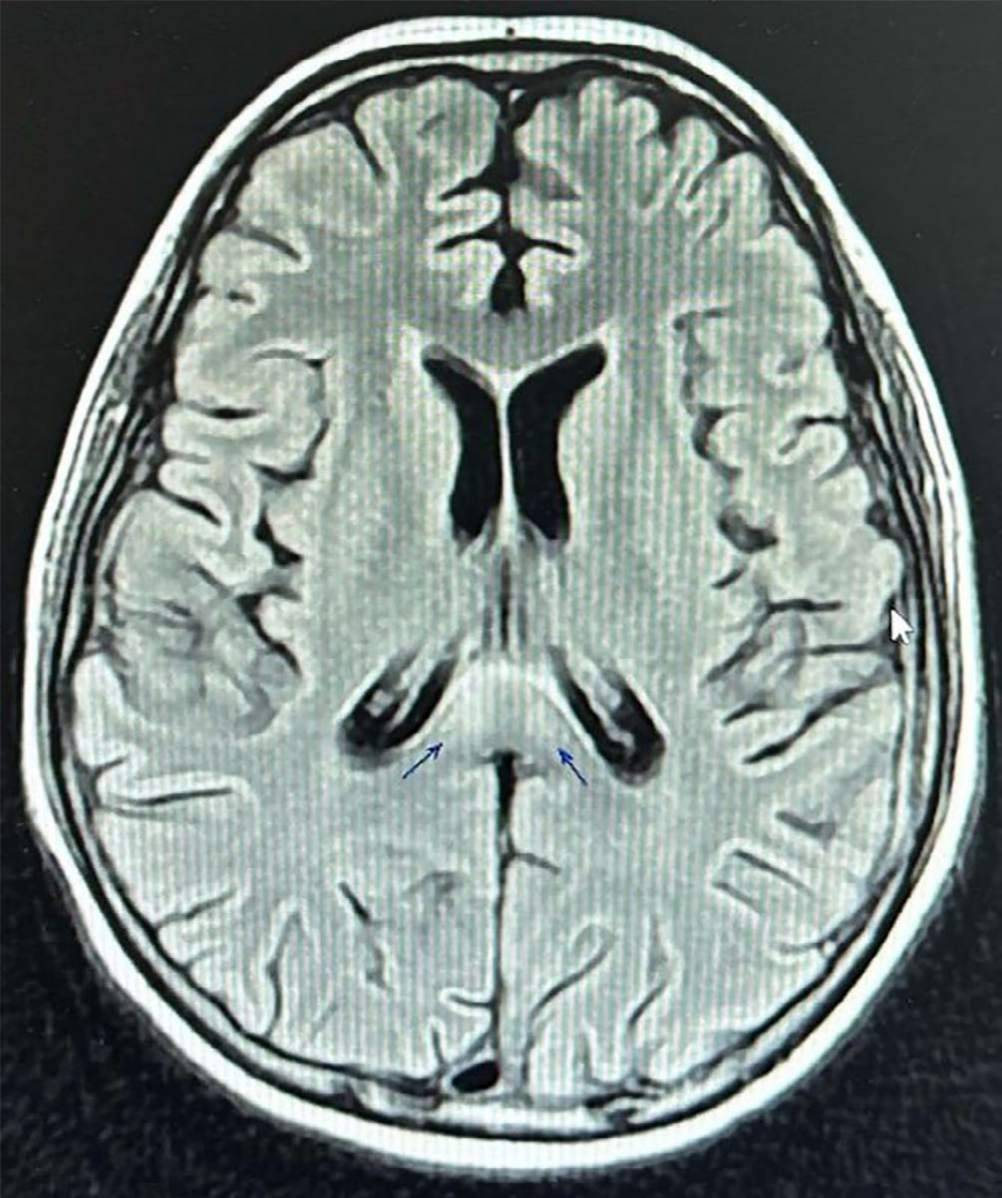
Brain MRI. The image shows two foci of altered signal intensity within the splenium of the corpus callosum (pointed with arrows) eliciting bright T2/FLAIR signal intensity with mild restriction in the DWIs, suggestive of cytotoxic lesions.

First, the induction chemotherapy was initiated (as shown in Table 2). Initially, the patient tolerated chemotherapy well for about 8 cycles of induction therapy, but he later developed progressive pain in the left inguinal area and lower limbs, eventually becoming unable to walk or carry weight. An electromyography/nerve conduction study revealed no response from the left and right superficial peroneal sensory nerves and the left sural sensory nerve from both lower limbs (Figures 2 and 3), confirming sensory polyneuropathy in both lower limbs, indicating chemotherapy‐induced neuropathy. The condition was managed with Gabapentin (300 mg, twice daily for 2 months), which alleviated the pain. A week later, an MRI of the brain revealed cytotoxic lesions in the corpus callosum (Figure 4), suggesting extensive chemotherapy‐induced neurotoxicity. The patient was placed under conservative management by a neurologist. At this stage, the decision to omit vincristine from the Berlin‐Frankfurt‐Munster (BFM) consolidation and interim maintenance phases of therapy was made.

Following completion of induction therapy, the patient developed neutropenia and vitamin D deficiency, requiring vitamin D supplementation (50 000 IU, once weekly) and careful monitoring for any signs of sepsis due to neutropenia. However, a bone marrow examination a week later was negative for minimal residual disease (MRD) and showed blast cells below 5%. The patient was then put on BFM consolidation therapy without vincristine as per the regimen shown in Table 2. He was also started on physiotherapy sessions to help him recover from his lower limb dysfunction. Then, consolidation therapy was followed by interim maintenance therapy (Table 2) which was initiated to sustain remission.

The patient was then discharged from the hospital and advised to attend regular follow‐ups and continue attending his weekly physiotherapy sessions for lower limb function for 2 months. During the follow‐up done in June 2024, the patient was found to have recovered from previous neurological deficits, with intact lower limb reflexes and sensations, and was walking normally with a normal gait, albeit at a slow pace.

Following the completion of interim maintenance therapy, as CIPN symptoms began to improve, the patient was given three courses of Blinatumomab, and we cautiously reintroduced vincristine during the delayed intensification course, starting at 50% of the standard dose and later escalating to full dose at 12‐week intervals to ensure complete remission. The patient tolerated vincristine well upon reintroduction, without complications. Tests done at the follow‐up visit in October 2024 revealed normal liver and renal function profiles.

By the December 2024 follow‐up, the patient's general condition had improved, with better mobility, and he was able to walk short distances with support. Laboratory results showed an improved hemoglobin level (9.6 g/dL) and platelet count (211 × 10^9^/L), with mild leucopenia (2.4 × 10^9^/L), marked absolute neutropenia (0.03 × 10^9^/L) and mild absolute monocytosis (1.07 × 10^9^/L). Cytology examination of cerebrospinal fluid at his most recent follow‐up in February 2025 showed no evidence of malignancy. Additionally, his CBC profile had improved, with resolution of neutropenia and monocytosis (neutrophil count: 1.46 × 10^9^/L, monocyte count: 0.16 × 10^9^/L) (Table 3). The patient therefore demonstrated a favorable outcome with successful remission of ALL, along with effective management of CIPN, which otherwise could have significantly impacted his quality of life.

**TABLE 3:** Timeline of events during the evaluation and management of the patient.

Date	Event
5/11/2023	Patient referred to our hospital. CBC and blood film analysis revealed severe anemia, absolute neutropenia, lymphocytosis, and the presence of 35% blast cells
6/11/2023	Flowcytometry results confirmed B‐cell ALL
9/11/2023–7/12/2023	Standard induction therapy (with vincristine) was given
2/12/2023	Patient developed progressive pain in lower limbs
3/12/2023	EMG/NCS confirmed sensory polyneuropathy in both lower limbs, suggesting CIPN
11/12/2023	Brain MRI revealed cytotoxic lesions in corpus callosum
13/12/2023	MRD assessment post‐induction (at day 35) was negative
25/12/2023	BFM consolidation therapy was initiated (without vincristine)
27/12/2023	Interim maintenance therapy was initiated (without vincristine)
25/12/2023–28/2/2024	Regular physiotherapy follow‐ups were attended
6/6/2024	Follow‐up visit: Recovery from previous neurological deficits, with intact lower limb reflexes and sensations. Patient was walking normally with normal gait, albeit at a slow pace.
8/7/2024	Delayed intensification course was initiated with vincristine reintroduction at 50% of the standard dose, followed by full dose at 12‐week intervals
15/10/2024	Follow‐up visit: Liver and renal function parameters were within normal range.
2/12/2024	Follow‐up visit: CBC showed improved hemoglobin and platelet count. However, mild leucopenia, marked absolute neutropenia and mild absolute monocytosis were noted.
3/2/2025	Last follow‐up. Improved CBC profile with resolution of neutropenia and monocytosis.
4/2/2025	CSF cytological examination showed no leukemic blast cells.

## 3 Discussion

CIPN in pediatric ALL poses significant challenges, often persisting long after chemotherapy ends, highlighting the need for effective interventions to improve long‐term functional performance and quality of life. However, limited understanding of CIPN mechanisms in children, coupled with complex, neurotoxic chemotherapy regimens, complicates the identification of specific drug target/s for intervention.

Among various drugs used in the treatment of ALL, vincristine is a cornerstone agent in both pediatric and adult cases. However, its well‐documented neurotoxicity led to the development of CIPN in our patient, necessitating its omission from the consolidation and interim maintenance phases. This modification of the standard chemotherapy regimen is what makes this case unique because the decision of omitting vincristine made this case particularly challenging, as its omission carried the risk of reduced treatment efficacy and an increased likelihood of relapse, as suggested by previous studies [6, 10].

With risk‐adapted therapies tailored to individual risk profiles of patients, it is possible to optimize outcomes while minimizing adverse effects. Studies show that detailed biological characterization of lymphoblasts, germline variability, and early treatment response can help identify ultra‐low‐risk patient subsets suitable for less intensive chemotherapies [11, 12]. For instance, the rs924607 polymorphism in the CEP72 gene is linked to VIPN, underscoring the role of genetic factors in personalized treatment [13]. Furthermore, regular evaluation of neuropathy, both clinically and electrophysiologically, can help in early detection and management of neuropathic symptoms in ALL survivors [6]. Thus, risk‐adapted individualized therapies can help prepare and monitor children who are at risk of CIPN, as well as provide essential care and advice to those with symptoms.

Vincristrine drug is most commonly associated with CIPN, while others like methotrexate, cytosine arabinoside, daunorubicin, etoposide, 6‐Mercaptopurine, and L‐asparaginase, rarely cause it [6]. CIPN predominantly manifests as sensory neuropathy that may accompany with motor and autonomic changes. These usually emerge from a week to months after completion of the chemotherapy. Children may experience paresthesia (tingling, numbness, prickling sensations or “stocking and glove” pain), and dysesthesia (hypersensitivity to touch or temperature changes), indicating sensory loss. These sensations can range from bothersome to disabling and may affect daily activities. Motor symptoms include weakness in the muscles of extremities. Some patients may exhibit autonomic symptoms, like changes in blood pressure, heart rate, or sweating patterns [14].

If left untreated, CIPN can severely impact a child's quality of life due to chronic neuropathic pain, impaired motor functions, and, in severe cases, neurological complications, like sensory ataxia, loss of autonomy, increasing the risk of falls and injuries [15]. Moreover, persistent symptoms may also hinder chemotherapy tolerance and response, potentially impacting the overall prognosis [16, 17]. Hence, early recognition and management of CIPN are essential to mitigate these complications and ensure the best possible care for pediatric ALL patients.

Currently, no national guidelines exist for the recognition or management of CIPN. The current symptom management often relies on dose reduction, interruption, or discontinuation of chemotherapy, significantly impacting survival. Therefore, managing CIPN requires a comprehensive approach to alleviate symptoms and improve quality of life. To date, duloxetine, a serotonin‐norepinephrine reuptake inhibitor in a dose of 60 mg/day, is the only approved drug for chronic CIPN pain. However, its potential drug–drug interactions must be carefully considered, especially with medications affecting serotonin levels or liver enzymes, which can impact its efficacy and compromise the patient's safety [17]. On the other hand, gabapentinoids like gabapentin and pregabalin have shown to alleviate both neuropathic pain and neurologic deficit with mild side effects [18], with pregabalin offering relief for patients unresponsive to gabapentin due to its superior pharmacokinetics [18]. A multicenter cohort study involving 127 children with neurological disorders and severe disabilities in palliative care found that gabapentin was associated with overall improvement in pain intensity [19]. However, a recent systematic review and meta‐analysis concluded that gabapentinoids do not significantly prevent CIPN in the preventive setting, and that in the treatment setting, they appear to offer some benefit, though further evidence is needed to confirm their effectiveness [20].

Non‐pharmacological therapies like physical therapy, massage, sensory integration, and therapeutic exercises have shown promise in relieving CIPN pain [21]. Sensorimotor training and whole‐body vibration exercises can reduce CIPN symptoms and improve motor and sensory deficits. Additionally, lifestyle modifications and patient‐reported outcomes may enhance self‐management [21]. Thus, a multidisciplinary approach with collaborative care involving oncologists, nurses, physical therapists, and mental health professionals should be considered to provide a comprehensive support for children with CIPN.

In this case report, our patient, a 16‐year‐old boy with ALL who developed CIPN during induction therapy, achieved complete remission with significant improvement in mobility and neurological functions with our risk‐mitigation individualized approach. Our approach to this case has several strengths. We prioritized a patient‐centered care strategy, carefully balancing the management of CIPN with effective ALL treatment. Regular follow‐ups and a multidisciplinary approach ensured comprehensive monitoring and management of CIPN. Our strategic risk‐mitigation strategy, which involved omission of vincristine in the consolidation and interim phases of therapy with its gradual reintroduction at a reduced dose (50%), followed by dose escalation at extended intervals during the delayed intensification phase, minimized neurotoxicity while ensuring complete remission. However, there are a few limitations. Although vincristine was successfully reintroduced later during the delayed intensification phase, its temporary omission posed an inherent risk of disease relapse, necessitating long‐term follow‐up to confirm sustained remission.

In conclusion, this case report highlights the significance of an individualized treatment approach in managing pediatric ALL complicated by CIPN. It also emphasizes the importance of timely recognition and management of CIPN following a multidisciplinary approach. The key lesson to take from this case is that balancing effective cancer treatment with the management of chemotherapy‐related side effects demands careful planning and flexibility, which should be based on the individual risk profiles of patients. Moreover, this case reinforces the importance of regular follow‐ups to monitor sustained remission and the effectiveness of CIPN management, ensuring optimal patient outcomes.

## Author Contributions

Investigation: E.A. and S.A. writing‐original draft: O.A.H. writing‐review and editing: E.A. and S.A.

## Ethics Statement

Ethical approval was not required for this study as it is a case report of a patient who received standard medical care in our hospital (NBK Children's Hospital, Kuwait) during his hospital admission. It is to be noted that NBK Children's Hospital is only a healthcare facility, and not an academic institution, and therefore, under Kuwait's law, ethical approval for such studies is not required. However, given the rarity of this case in the medical literature, we sought and obtained written informed consent from the guardians of the pediatric patient for the purpose of publication.

## Conflicts of Interest

The authors declare no conflicts of interest.

## Supporting information


**Figure S1:** The dot plot shows distinct clustering of leukocyte populations based on CD45 expression and side scatter characteristics (SSC). A well‐defined population with dim CD45 and low SSC, gated as blasts (red), accounted for 43.2% of total events. This immunophenotypic pattern was consistent with an immature blast population typically seen in acute leukemia. Mature lymphocytes (green; bright CD45, low SSC) (37.8%), monocytes (blue; intermediate CD45 and SSC) (0.4%), and granulocytes (purple and orange; dim CD45, high SSC) (7.8% + 6.5%) were identified in their respective regions.
Immunophenotypic subsets further suggested a precursor B‐cell ALL phenotype, as supported by the high percentage of CD10 + CD19+ (88.9% of gated blasts), CD10+ (88.8%), and CD19+ (92.4%) events.
**Figure S2:** The dot plot shows distinct clustering of leukocyte populations based on CD45 expression and side scatter characteristics (SSC). A population with dim CD45 and low SSC, gated as blasts (red), accounted for 40% of total events, consistent with acute leukemia profile. Mature lymphocytes (green; bright CD45, low SSC) (39.3%), monocytes (blue; intermediate CD45 and SSC) (0.4%), and granulocytes (purple; dim CD45, high SSC) (9.2%) were identified in their respective regions.
Immunophenotypic subsets further showed high percentage of CD20+ (75.6% of gated blasts) while low percentage of CD34‐ (3.9%) events.
**Figure S3:** This CD45/SSC‐A dot plot shows a population of leukocyte clustered with dim CD45 expression and low SSC, gated as blasts (red), accounting for 54.3% of total events. Mature lymphocytes (green; bright CD45, low SSC) (13.9%), monocytes (blue; intermediate CD45 and SSC) (0.5%), and granulocytes (purple; dim CD45, high SSC) (4.9%) were identified in their respective regions.
Immunophenotypic subsets further showed positive expression of TdT+ (73.8% of gated blasts) while negative expression of myeloperoxidase (MPO‐) (0.1%) events.
**Figure S4:** This CD45/SSC‐A dot plot shows a population of leukocyte clustered with dim CD45 expression and low SSC, gated as blasts (red), accounting for 42.6% of total events. Mature lymphocytes (green; bright CD45, low SSC) (41.6%), monocytes (blue; intermediate CD45 and SSC) (0.3%), and granulocytes (purple; dim CD45, high SSC) (6.2%) were identified in their respective regions.
Immunophenotypic subsets further showed positive expression of HLA‐DR+ (97.9% of gated blasts) while negative expression of CD33‐ (0.2%) events.

## Data Availability

Data sharing is not applicable to this article as no new data were created or analyzed in this study.
