# Supplementary material for: Chemotherapy‐Induced Peripheral Polyneuropathy in Pediatric Acute Lymphoblastic Leukemia: A Case Report on Manifestation, Management, and Outcome
Source: Cancer Rep (Hoboken). 2025 Aug 20;8(8):e70293. doi: 10.1002/cnr2.70293 (PMC12365659; doi:10.1002/cnr2.70293)
Supplement: Supplementary file 1 — Figure S1: The dot plot shows distinct clustering of leukocyte populations based on CD45 expression and side scatter characteristics (SSC). A well‐defined population with dim CD45 and low SSC, gated as blasts (red), accounted for 43.2% of total events. This immunophenotypic pattern was consistent with an immature blast population typically seen in acute leukemia. Mature lymphocytes (green; bright CD45, low SSC) (37.8%), monocytes (blue; intermediate CD45 and SSC) (0.4%), and granulocytes (purple and orange; dim CD45, high SSC) (7.8% + 6.5%) were identified in their respective regions. Immunophenotypic subsets further suggested a precursor B‐cell ALL phenotype, as supported by the high percentage of CD10 + CD19+ (88.9% of gated blasts), CD10+ (88.8%), and CD19+ (92.4%) events. Figure S2: The dot plot shows distinct clustering of leukocyte populations based on CD45 expression and side scatter characteristics (SSC). A population with dim CD45 and low SSC, gated as blasts (red), accounted for 40% of total events, consistent with acute leukemia profile. Mature lymphocytes (green; bright CD45, low SSC) (39.3%), monocytes (blue; intermediate CD45 and SSC) (0.4%), and granulocytes (purple; dim CD45, high SSC) (9.2%) were identified in their respective regions. Immunophenotypic subsets further showed high percentage of CD20+ (75.6% of gated blasts) while low percentage of CD34‐ (3.9%) events. Figure S3: This CD45/SSC‐A dot plot shows a population of leukocyte clustered with dim CD45 expression and low SSC, gated as blasts (red), accounting for 54.3% of total events. Mature lymphocytes (green; bright CD45, low SSC) (13.9%), monocytes (blue; intermediate CD45 and SSC) (0.5%), and granulocytes (purple; dim CD45, high SSC) (4.9%) were identified in their respective regions. Immunophenotypic subsets further showed positive expression of TdT+ (73.8% of gated blasts) while negative expression of myeloperoxidase (MPO‐) (0.1%) events. Figure S4: This CD45/SSC‐A dot plot shows a popul [file CNR2-8-e70293-s001.docx]

**SUPPLEMENTARY MATERIAL**

**CD45 vs SSC-A dot plots from flow cytometric analysis of peripheral blood showing immunophenotypic distribution of leukocyte populations**

**
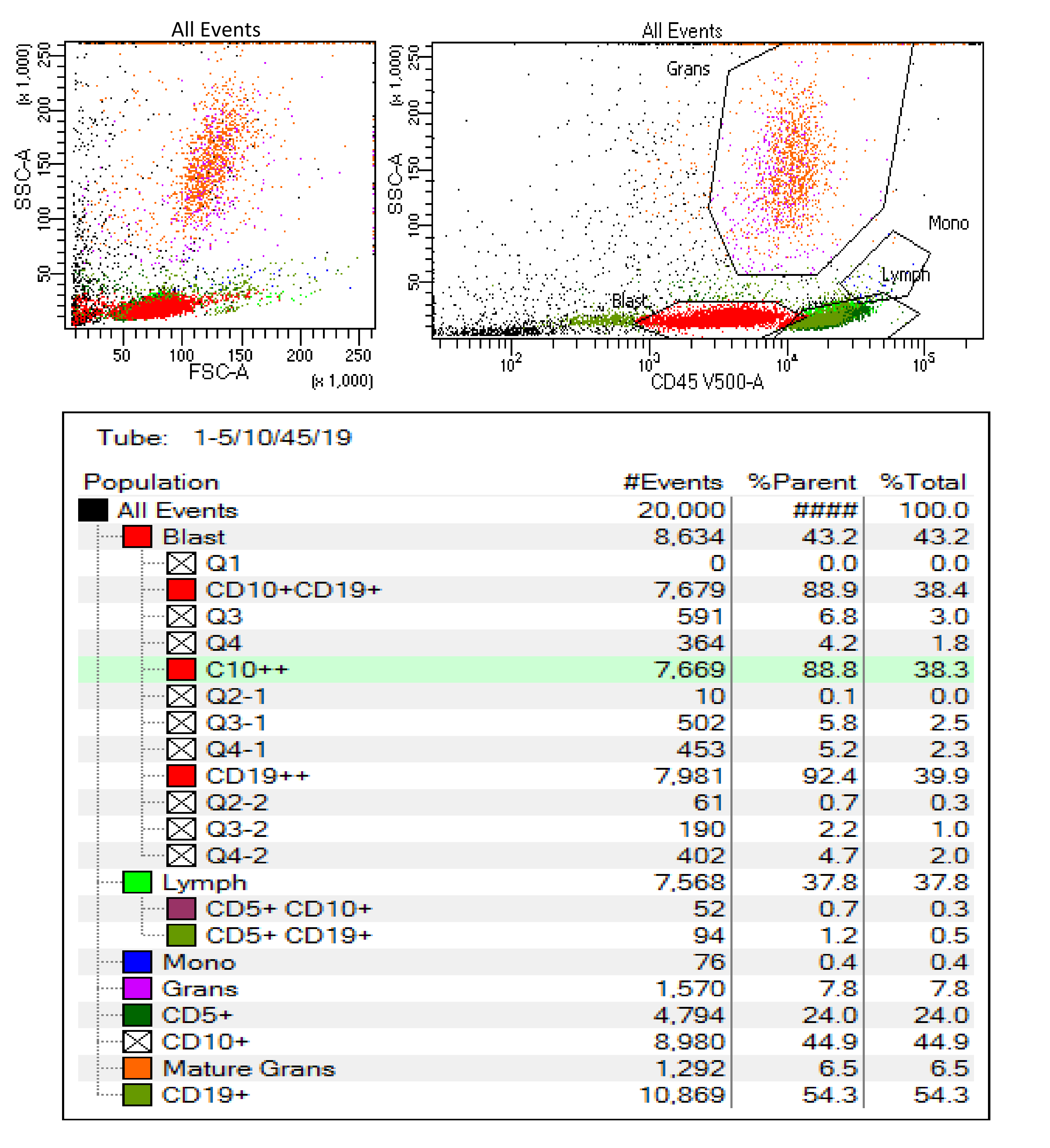
**

**Figure S1:** The dot plot shows distinct clustering of leukocyte populations based on CD45 expression and side scatter characteristics (SSC). A well-defined population with dim CD45 and low SSC, gated as blasts (red), accounted for 43.2% of total events. This immunophenotypic pattern was consistent with an immature blast population typically seen in acute leukemia. Mature lymphocytes (green; bright CD45, low SSC) (37.8%), monocytes (blue; intermediate CD45 & SSC) (0.4%), and granulocytes (purple and orange; dim CD45, high SSC) (7.8%+6.5%) were identified in their respective regions.

Immunophenotypic subsets further suggested a precursor B-cell ALL phenotype, as supported by the high percentage of CD10+CD19+ (88.9% of gated blasts), CD10+ (88.8%), and CD19+ (92.4%) events.

**
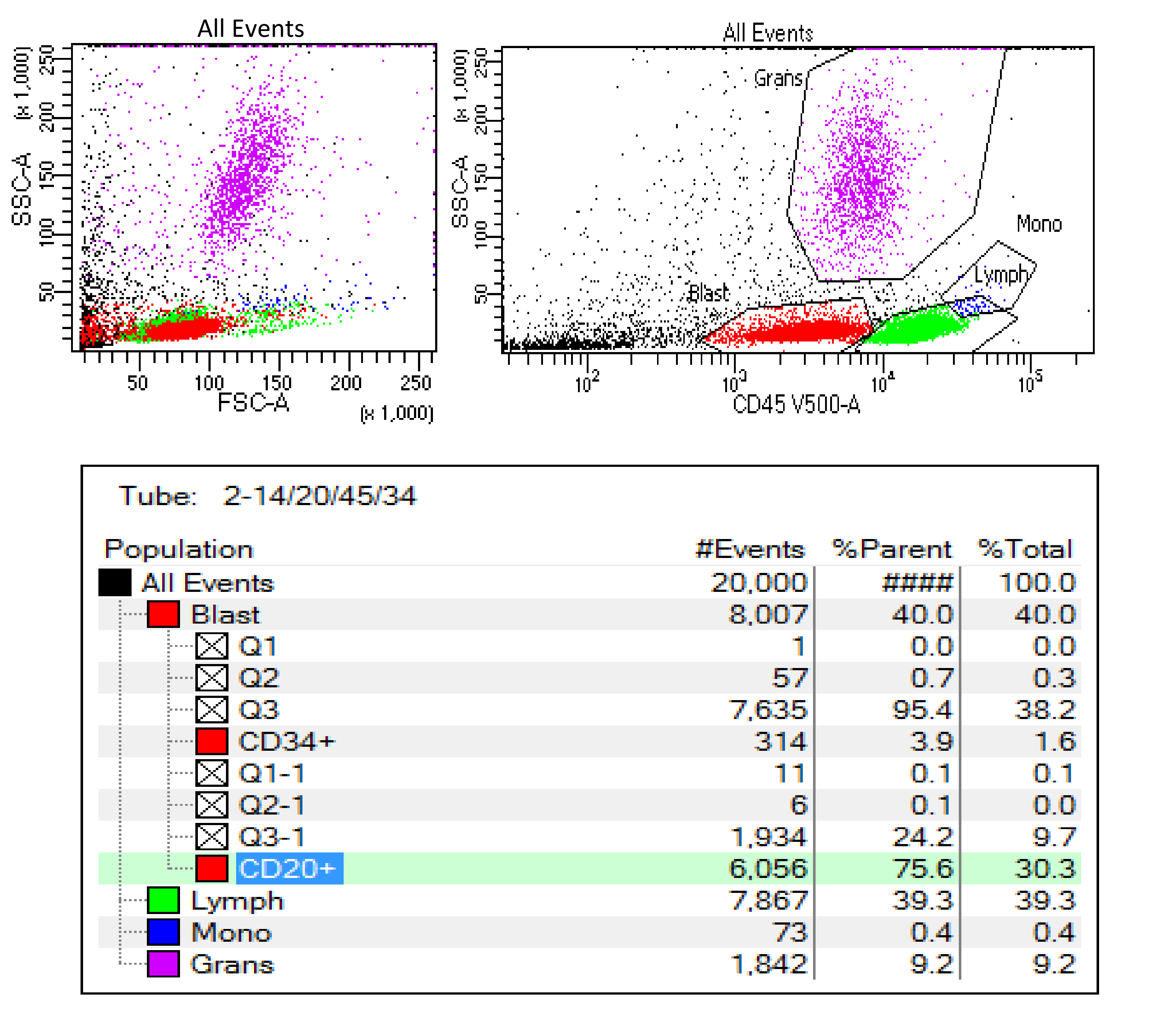
**

**Figure S2:** The dot plot shows distinct clustering of leukocyte populations based on CD45 expression and side scatter characteristics (SSC). A population with dim CD45 and low SSC, gated as blasts (red), accounted for 40% of total events, consistent with acute leukemia profile. Mature lymphocytes (green; bright CD45, low SSC) (39.3%), monocytes (blue; intermediate CD45 & SSC) (0.4%), and granulocytes (purple; dim CD45, high SSC) (9.2%) were identified in their respective regions.

Immunophenotypic subsets further showed high percentage of CD20+ (75.6% of gated blasts) while low percentage of CD34- (3.9%) events.

**
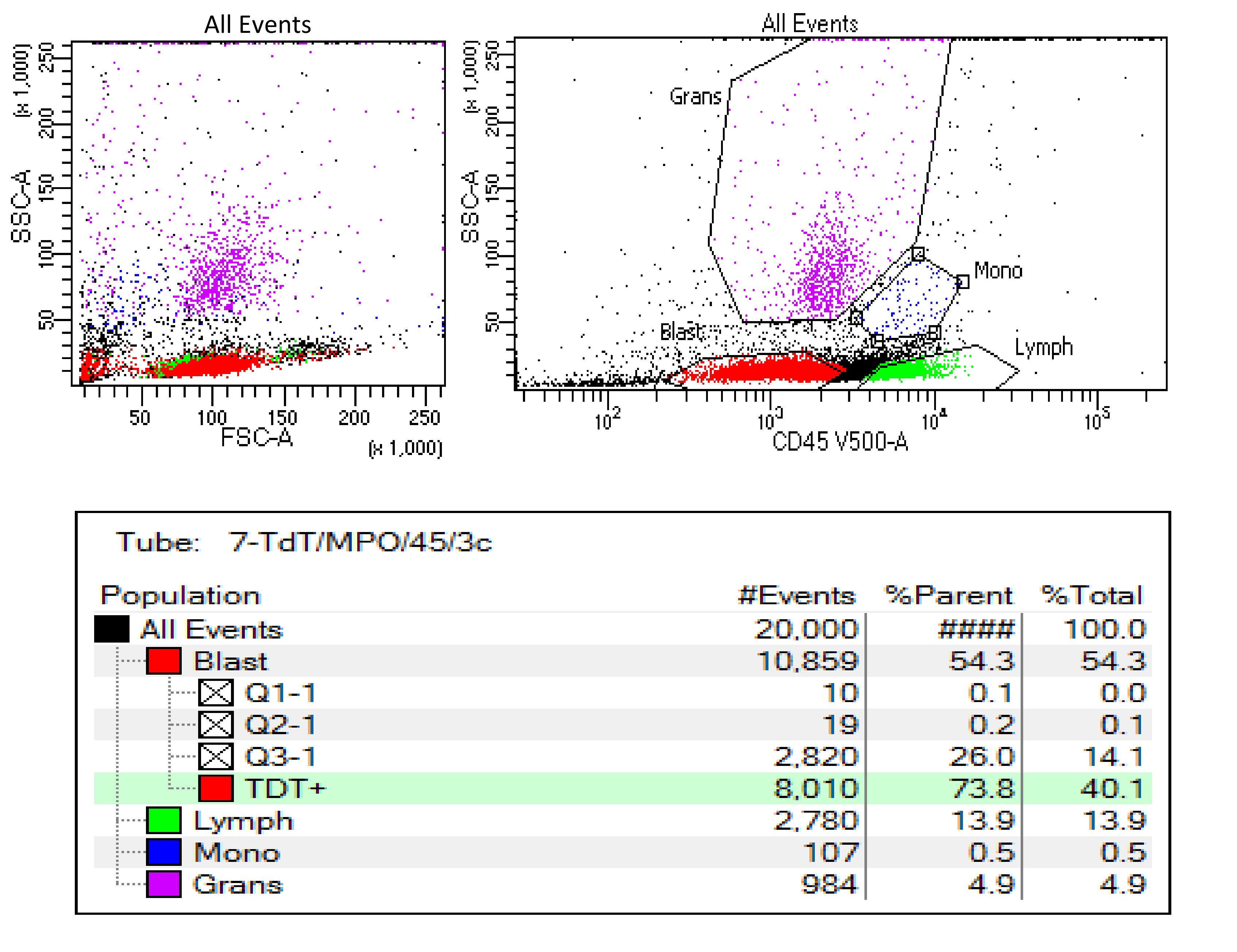
**

**Figure S3:** This CD45/SSC-A dot plot shows a population of leukocyte clustered with dim CD45 expression and low SSC, gated as blasts (red), accounting for 54.3% of total events. Mature lymphocytes (green; bright CD45, low SSC) (13.9%), monocytes (blue; intermediate CD45 & SSC) (0.5%), and granulocytes (purple; dim CD45, high SSC) (4.9%) were identified in their respective regions.

Immunophenotypic subsets further showed positive expression of TdT+ (73.8% of gated blasts) while negative expression of myeloperoxidase (MPO-) (0.1%) events.

**
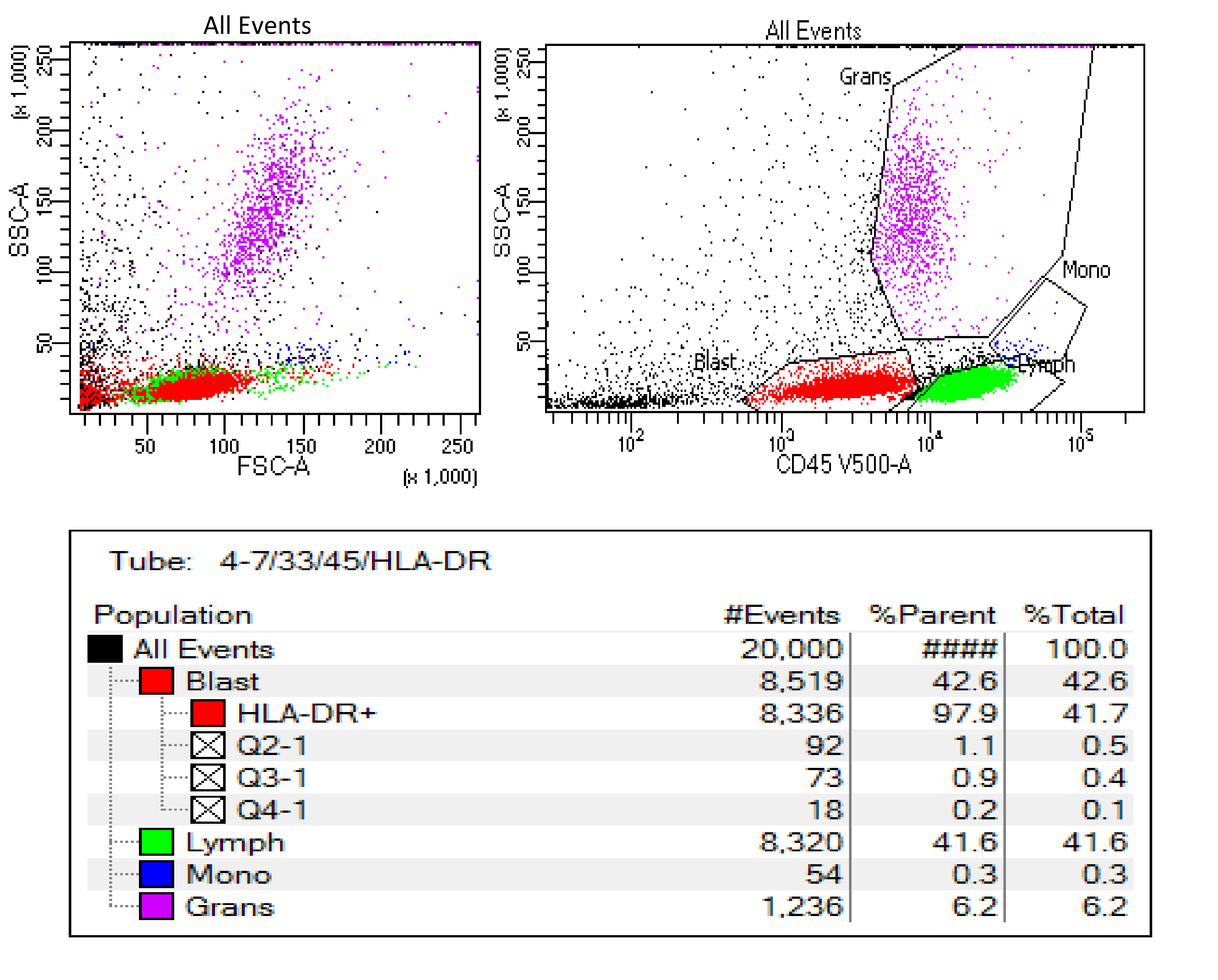
**

**Figure S4:** This CD45/SSC-A dot plot shows a population of leukocyte clustered with dim CD45 expression and low SSC, gated as blasts (red), accounting for 42.6% of total events. Mature lymphocytes (green; bright CD45, low SSC) (41.6%), monocytes (blue; intermediate CD45 & SSC) (0.3%), and granulocytes (purple; dim CD45, high SSC) (6.2%) were identified in their respective regions.

Immunophenotypic subsets further showed positive expression of HLA-DR+ (97.9% of gated blasts) while negative expression of CD33- (0.2%) events.
